# Preventive Effects of Pomegranate Seed Extract on Bleomycin-Induced Pulmonary Fibrosis in Rat

**Published:** 2013-05-04

**Authors:** Ali Asghar Hemmati, Annahita Rezaie, Pegah Darabpour

**Affiliations:** 1 School of Pharmacy, Ahvaz Jundishapur University of Medical Sciences, Ahvaz, IR Iran; 2 Department of Pathology, Faculty of Veterinary Medicine, Shahid Chamran University, Ahvaz, IR Iran; 3 School of Pharmacy, Ahvaz Jundishapur University of Medical Sciences, Ahvaz, IR Iran

**Keywords:** Pulmonary Fibrosis, Bleomycin, Antioxidants

## Abstract

**Background:**

Oxidative stress plays an important role in the pathogenesis of bleomycin-induced lung fibrosis and many antioxidant agents have been used for the treatment of this disease in animals.

**Objectives:**

To evaluate the antioxidant effects of pomegranate seed extract (PSE) on bleomycin treated rats.

**Materials and Methods:**

Male Spraque – Dawley rats were divided into 5 groups: rats in groups I (bleomycin) and II (control) were given a single dose of bleomycin (7.5 IU/kg, intratracheally) in the bleomycin group and same amount of saline in the control, respectively. Treatment groups (III-V) were given PSE (100,200,400 mg/kg) orally a week before the bleomycin injection and this was continued for 3 weeks. At day 28, animals were sacrificed and lungs were removed for histological investigation.

**Results:**

Histological analysis showed that PSE could prevent pathological changes that were seen in the bleomycin group.

**Conclusions:**

Results of the present study showed that hydroalcoholic extracts of pomegranate seeds had a significant protective effect against bleomycin-induced lung fibrosis by its antioxidant properties. The highest protective effect was observed for the 400 mg/kg dose.

## 1. Background

Oxidative stress is defined as an inequality between the production of free radicals and the ability of antioxidant defenses to scavenge them. Oxidative stress is one of the major mechanisms involved in the Pathogenesis of pulmonary fibrosis (1). It is believed that pulmonary fibrosis (PF) is the result of pathophysiological reactions to injury that can be caused by stimulants such as radiation, infection, drugs, and being exposed to toxic particles such as silica and asbestos. PF is characterized by alveolar epithelial cell injury, infiltration of inflammatory cells such as neutrophils and macrophages and differentiation of fibroblasts to myofibroblasts. These events, consequently, result in collagen deposition and changes in the lung structure that bring about reduced gas exchange and lessened pulmonary compliance. PF in patients usually do not improve significantly with the available treatments, although the treatments may slow disease progression. Despite the fact that PF is one of the deadly illnesses, there are hardly any therapeutic choices available and finding new healing agents is vital for the treatment of PF. According to the role of oxidative stress in PF, utilization of antioxidant agents for the treatment of the disease appears to be logical. Bleomycin (BLM) is an anti-cancer agent, which has limited therapeutic application in clinics because of its dose-dependent toxicity. Bleomycin-induced pulmonary fibrosis is a commonly used animal model of pulmonary fibrosis (2). *Punica granatum L* (Punicaceae), generally referred to as pomegranate, is a Mediterranean small tree. Numerous studies have been conducted on pomegranate because of its antioxidant effects. Recently, the high antioxidant activity of the extracts from the various parts of pomegranate fruit including peel, juice and seeds has been shown .This antioxidant activity has been said to be the result of a high level of phenolic compounds (3-5). Pomegranate is reported to have significant amounts of phenolic compounds, such as anthocyanins (3-glucosides and 3,5-diglucosides of delphinidin, cyanidin, and pelargonidin), ellagic acid, punicalin, punicalagin, pedunculagin and different flavanols (6). Researchers have demonstrated that pomegranate is a potent antioxidant (6-8) anti-inflammatory (9) antidiabetic (10) and neuroprotective (11).

## 2. Objectives

Based on the aforementioned results, the present study attempts to find the possible protective effects of pomegranate seed extract on bleomycin-induced pulmonary fibrosis by using a semi-quantitative assessment of lung histology.

## 3. Materials and Methods

### 3.1. Reagents

Bleomycin (bleocin) was purchased from kakaya Ltd, China. Pomegranate (*Punica granatum*), was also purchased from a local market in Ahvaz.

### 3.2. Hydro-alcoholic Extract of Pomegranate Seed

Pomegranate juice was removed by pressing. Seeds were allowed to dry in shade. Dried seeds were ground to fine powder by a grinder. About 100 g of the powder was mixed with 300 ml of 70% ethanol in distilled water and kept for 3 days at room temperature. The extract was then filtered. Solvent (ethanol / water) was removed using a rotary evaporator under vacuum at 50 ˚C. Dried extract obtained and kept in the refrigerator. Enough amounts of the dried extract were suspended in water and administered to animals of the treatment groups.

### 3.3. Animal Groups and Treatment

Male Sprague – Dawley rats (190–220 g) were purchased from the animal house and research center of the Jundishapur University of Medical Sciences, Ahvaz, Iran. All animals were maintained under a 12:12 h light–dark cycle at 23 ˚C with food and water available ad libitum for at least 1 week before starting the experiments. The study protocol was in accordance with the guidelines for the care and use of laboratory animals. All rats were randomly assigned into either the saline group or the BLM treated group, or the three treatment groups at a dose of 100, 200 or 400 mg/kg body weight of PSE. PSE was prepared daily just before application to the rats and was administered orally by gavage needle. Pulmonary fibrosis was induced by endotracheal injection of BLM at the dose of 7.5 IU/kg body weight except in the saline group. Saline group received equal amounts of 0.9% saline. Fibrosis was assessed by lung histology. On each day during the experimental period, rats in the PSE groups were administered PSE by gavage, and the saline rats and BLM rats received equal amounts of 0.9% saline, respectively. First dose of PSE and vehicle were given 1 week before the BLM injection and continued until sacrifice.

### 3.4. Histological Examination

At the end of the experiment, animals were sacrificed and the lungs were dissected out. Lungs were fixed with 10% buffered formalin. The lung specimens were dehydrated and embedded in paraffin. For histological examination, firstly, 4 μm sections of fixed embedded tissues were cut on a rotary microtome (Leica model 2235, Germany), and then placed on glass slides, de-paraffinized, and stained with hematoxylin and eosin. All sections were studied using light microscopy. A semi-quantitative grading of the staining was used for the evaluation of inflammation and fibrosis with a scoring system from 0 to 3+ (0: normal, +: presence of inflammation and fibrosis involving less than 25% of the lung parenchyma, ++: lesions involving 25–75% of the lung, moderate thickening of bronchial wall and formation of fibrous bands or small fibrous mass, + + +: lesions involving more than 75% of the lung, severe distortion of structure and fibrous obliteration of fields).

## 4. Results 

**Figure 1.**
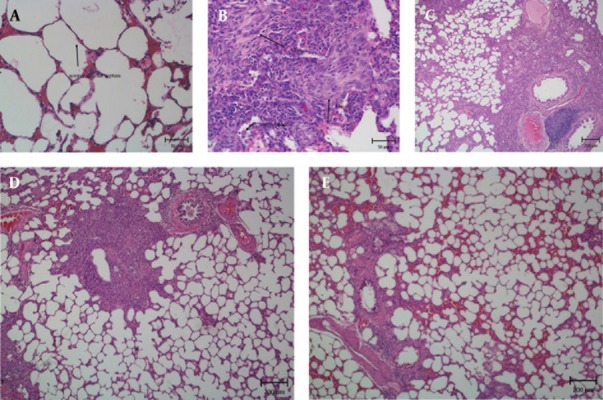
The Influence of PSE on Histopathological Changes in Rats with BLM-induced Lung Injury. **A.** Normal Lung Histology in the Saline Group; **B.** Inflammatory Cell Infiltration and Fibrosis in the BLM Group; **C.** Weaker Fibrosis in the PSE Group (100 mg/kg); **D.** Reduced Lung Fibrosis in the PSE Group (200 mg/kg); **E.** Marked Prevention of Fibrosis in the PSE Group (400 mg/kg).

The histopathological evaluation of the lung tissues from either saline, BLM and PSE treated groups revealed that sections taken from the saline group had a normal structure without any pathologic changes when scrutinized under a light microscope. Lungs of saline rats displayed normal alveolar spaces and normal thickening of alveolar septa (Figure 1A). The lungs of rats treated with BLM showed infiltration of inflammatory cells in alveolar spaces, increasing the thickness of the intra-alveolar septa, and accumulation of collagen and fibrosis (Figure 1B). PSE treatment could help prevent some of the pathological changes induced by BLM. There was less inflammatory cell infiltration and reduced collagen deposition in PSE groups. Relative reductions in lung fibrosis and collagen fibers were observed in the group treated with a low dose of 100mg/kg PSE (Figure 1C). Significant reductions of collagen deposition and lung fibrosis were observed in the group treated with higher dose of PSE (200 mg/kg) (Figure 1D). The group that received the highest dose (400 mg/kg) showed the greatest improvement similar to the saline group. The extent of inflammation was significantly less severe compared with that in the BLM group (Figure 1E). PSE could diminish the severity of lung damage. The grades of fibrosis in the five groups are presented in Table. The semi-quantitative assessment of lung sections showed that bleomycin treatment caused an increase in the pathology score when compared to the saline group. PSE treatment prevented fibrosis and lowered the pathology score in the treatment groups.

**Table.** Severity of Histological Changes in Treatment Groups

Name of group	Severity of lesion
**Saline Treated**	0
**BLM (Tositive Tontrol)**	+++
**PSE 100 mg/kg**	++
**PSE 200 mg/kg**	++
**PSE 400 mg/kg**	+

## 5. Discussion

The results of the present study demonstrate that the antioxidant effects of PSE could prevent the accumulation of collagen and lung pathological changes in a pulmonary fibrosis rat model. As mentioned before, pulmonary fibrosis is characterized by the infiltration of inflammatory cells, fibroblasts differentiation, and extracellular matrix remodeling and collagen deposition. It has been described that activated inflammatory cells accumulated in the site of lung injury, release reactive oxygen species (ROS) that lead to an increase of fibroblasts in alveolar walls and lung fibrosis (12). The activated fibroblasts generate increased amounts of extracellular matrix proteins that interfere with the normal lung architecture and disable gas exchange in the lungs (13). Severe organ injury is caused by BLM administration which is the result of increased reactive oxygen species. ROS generated from BLM complexes with iron and damages important macromolecules like proteins, lipids, and DNA (14, 15). Due to the diminished BLM inactivating enzymes in the lungs, pulmonary injury as a reaction to systemic BLM treatment is often seen (16). Free radicals produced by BLM can cause damage to epithelial and endothelial cells in lung tissue. The initial damage in the lung leads to the infiltration of activated inflammatory cells into the lung parenchyma and release of different cytokines such as TGF-β and TNF-α. These eventually result in collagen deposition in the lung tissue. Oxidative stress is generally explained as the situation where there is no balance between ROS production and antioxidant defenses. This is a potential target for the development of therapeutics for the treatment of lung fibrosis. Many efforts have been made to eliminate the free radicals by using different types of antioxidants, but a cure for this disease has not yet been found. Flavonoids are one of the compounds, which have significant antioxidant properties and are capable of relative protection against injuries resulting from free radicals. In the present study we have tried to evaluate the antioxidant effects of hydroalcoholic pomegranate seed extracts and its therapeutic effects on fibrosis. Some plants that have polyphenolic compounds, such as Ginkgo biloba extracts and curcumin, have been reported to prevent lung oxidative stress and fibrosis in rats treated with BLM (17, 18). The marked antioxidant activity of pomegranate seed extracts seems to be due to the existence of polyphenols, which can release electrons that can react with free radicals to convert them to more stable product and end free radical chain reactions (4). Findings of this study show that antioxidant effects of hydroalcoholic extract of pomegranate seed in rat pulmonary fibrosis is dose-dependent and the best antioxidant effect was found to be in the highest dose of PSE (400 mg/kg). The histological findings of the current research certainly point out that PSE noticeably reduces the extent and severity of the histological signs of tissue damage. These observations are also confirmed by the semi-quantitative assessment. The exact mechanisms of PSE compounds may not have been covered by this study but in relation with findings of other studies we may make some suggestions. According to the oxidative stress hypothesis of pulmonary fibrosis, it has been suggested that PSE, a strong antioxidant, will be effective in preventing tissue pathological changes through its anti-fibrotic activity by scavenging ROS in the BLM rat model. The results of this study support the hypothesis that, PSE (probably due to the presence of polyphenols) can neutralize oxidative stress and scavenge free radicals, regardless of its anti-inflammatory effects. Therefore, PSE may help protect against lung fibrosis. Due to the high histopathological similarity between these experimental animal models and pulmonary fibrosis in humans, instead of using corticosteroids and immune suppressants, which have more side effects, we can use PSE as a natural drug for the management of pulmonary fibrosis. We suggest that in the future, more studies should be done with the use of this compound in patients.

## References

[1] McCord JM, Fridovich I (1978). The biology and pathology of oxygen radicals.. Ann Intern Med..

[2] Yildirim Z, Turkoz Y, Kotuk M, Armutcu F, Gurel A, Iraz M (2004). Effects of aminoguanidine and antioxidant erdosteine on bleomycin-induced lung fibrosis in rats.. Nitric Oxide..

[3] Aviram M, Dornfeld L, Rosenblat M, Volkova N, Kaplan M, Coleman R (2000). Pomegranate juice consumption reduces oxidative stress, atherogenic modifications to LDL, and platelet aggregation: studies in humans and in atherosclerotic apolipoprotein E-deficient mice.. Am J Clin Nutr..

[4] Singh RP, Chidambara Murthy KN, Jayaprakasha GK (2002). Studies on the antioxidant activity of pomegranate (Punica granatum) peel and seed extracts using in vitro models.. J Agric Food Chem..

[5] Gil MI, Tomas-Barberan FA, Hess-Pierce B, Holcroft DM, Kader AA (2000). Antioxidant activity of pomegranate juice and its relationship with phenolic composition and processing.. J Agric Food Chem..

[6] González-Molina E, Moreno DA, García-Viguera C (2009). A new drink rich in healthy bioactives combining lemon and pomegranate juices.. Food Chemistry..

[7] Plumb GW, de Pascual-Teresa S, Santos-Buelga C, Rivas-Gonzalo JC, Williamson G (2002). Antioxidant properties of gallocatechin and prodelphinidins from pomegranate peel.. Redox Rep..

[8] Seeram NP, Adams LS, Henning SM, Niu Y, Zhang Y, Nair MG (2005). In vitro antiproliferative, apoptotic and antioxidant activities of punicalagin, ellagic acid and a total pomegranate tannin extract are enhanced in combination with other polyphenols as found in pomegranate juice.. J Nutr Biochem..

[9] Ahmed S, Wang N, Hafeez BB, Cheruvu VK, Haqqi TM (2005). Punica granatum L. extract inhibits IL-1beta-induced expression of matrix metalloproteinases by inhibiting the activation of MAP kinases and NF-kappaB in human chondrocytes in vitro.. J Nutr..

[10] Huang TH, Peng G, Kota BP, Li GQ, Yamahara J, Roufogalis BD (2005). Anti-diabetic action of Punica granatum flower extract: activation of PPAR-gamma and identification of an active component.. Toxicol Appl Pharmacol..

[11] Loren DJ, Seeram NP, Schulman RN, Holtzman DM (2005). Maternal dietary supplementation with pomegranate juice is neuroprotective in an animal model of neonatal hypoxic-ischemic brain injury.. Pediatr Res..

[12] Weissler JC (1989). Idiopathic pulmonary fibrosis: cellular and molecular pathogenesis.. Am J Med Sci..

[13] Adamson IY, Bowden DH (1974). The pathogenesis of bloemycin-induced pulmonary fibrosis in mice.. Am J Pathol..

[14] Ishida R, Takahashi T (1975). Increased DNA chain breakage by combined action of bleomycin and superoxide radical.. Biochem Biophys Res Commun..

[15] Cunningham ML, Ringrose PS, Lokesh BR (1983). Bleomycin cytotoxicity is prevented by superoxide dismutase in vitro.. Cancer Lett..

[16] Muller WE, Zahn RK (1976). Determination of the bleomycin-inactivating enzyme in biopsies.. Gann..

[17] Daba MH, Abdel-Aziz AA, Moustafa AM, Al-Majed AA, Al-Shabanah OA, El-Kashef HA (2002). Effects of L-carnitine and ginkgo biloba extract (EG b 761) in experimental bleomycin-induced lung fibrosis.. Pharmacol Res..

[18] Venkatesan N, Punithavathi V, Chandrakasan G (1997). Curcumin protects bleomycin-induced lung injury in rats.. Life sci..

